# Innovative advances and future perspectives in injectable hydrogels for wound healing: a comprehensive review

**DOI:** 10.1186/s12938-026-01541-6

**Published:** 2026-04-30

**Authors:** Shohreh Fahimirad, Zohreh Ghazi Tabatabaei, Mohammad Reza Farahpour, Fatemeh Mahmoudian

**Affiliations:** 1 Molecular and Medicine Research Center, Arak University of Medical Sciences, Arak, Iran; 2 Clinical Research Development Unit of Amiralmomenin Hospital, Arak University of Medical Sciences, Arak, Iran; 3 Clinical Research Development Unit of Amirkabir Hospital, Arak University of Medical Sciences, Arak, Iran; 4 Department of Chemistry, Ah.C., Islamic Azad University, Ahar, Iran; 5 Social Determinants of Health Research Center, Semnan University of Medical Sciences, Semnan, Iran; 6 Cancer Research Center, Semnan University of Medical Sciences, Semnan, Iran; 7 Department of Clinical Sciences, Faculty of Veterinary Medicine, Ur.C., Islamic Azad University, Urmia, Iran

**Keywords:** Injectable hydrogel, In situ gelation, Wound healing, Controlled drug delivery, Regenerative biomaterials

## Abstract

Injectable hydrogels constitute a highly versatile and promising class of biomaterials for therapeutic use in both acute and chronic wounds. Engineered to mimic the structural and functional attributes of the native extracellular matrix (ECM), these hydrogels form a biomimetic, hydrated three-dimensional network that facilitates critical wound healing processes, such as cellular infiltration, angiogenesis, and extracellular matrix remodeling. Composed of a broad spectrum of biocompatible polymers, including naturally derived polysaccharides, such as alginate, hyaluronic acid, and chitosan, along with various synthetic polymers such as polyethylene glycol and polyvinyl alcohol injectable hydrogels can be precisely tailored in terms of viscoelastic properties, degradation kinetics, and bio-functionalization to meet specific clinical requirements. Their minimally invasive administration through a syringe or catheter, combined with in situ gelation triggered by physiological stimuli, such as pH, temperature, or ionic strength, allows conformal adaptation to complex wound geometries while minimizing surgical trauma. Furthermore, these hydrogels serve as adaptable scaffolds for the spatial and temporal controlled delivery of therapeutic agents, including growth factors, antimicrobial compounds, stem cells, and extracellular vesicles, enabling dynamic modulation of the wound microenvironment. Such functionalities facilitate regulated inflammation, oxidative stress mitigation, and tissue regeneration. Despite their substantial potential, challenges persist regarding mechanical stability under physiological load, immunomodulatory capacity, and regulatory pathways for clinical translation. Recent advancements—such as the integration of nanostructured components, stimuli-responsive crosslinking mechanisms, and bio-orthogonal chemistries—have expanded the functional capabilities of injectable hydrogels and improved their therapeutic efficacy. This review offers a comprehensive analysis of the present status and future directions of injectable hydrogel systems for wound healing, emphasizing innovative material strategies, delivery mechanisms, and translational hurdles. These insights highlight the critical role of injectable hydrogels in advancing the development of next-generation, precision-guided wound management technologies.

## Introduction

Disruption of the skin and underlying tissues, caused by mechanical trauma, chemical or thermal injury, microbial invasion, or immune dysregulation, initiates complex cellular and molecular cascades characterized by extracellular matrix (ECM) damage, vascular disruption, and activation of inflammatory and reparative signaling pathways [1, 2].

Chronic wounds, including diabetic foot ulcers, venous leg ulcers, and pressure injuries, represent a major clinical and socioeconomic burden worldwide. In these conditions, healing is arrested due to systemic and local pathophysiological factors, such as impaired angiogenesis, hypoxia, immune dysfunction, and defective collagen remodelling—most prominently observed in diabetes mellitus [3, 4]. Persistent microbial colonization and biofilm formation further exacerbate inflammation and disrupt regenerative signaling, underscoring the need for advanced therapeutic strategies capable of restoring coordinated healing responses [5, 6].

Hydrogel-based wound dressings have evolved from passive moisture-retaining materials into multifunctional biomaterials that actively modulate the wound microenvironment. Hydrogels consist of hydrophilic polymer networks capable of retaining large volumes of water while maintaining structural integrity. Both natural polymers (e.g., alginate, chitosan, hyaluronic acid, and gelatin) and synthetic polymers (e.g., polyethylene glycol, polyvinyl alcohol, and poloxamers) are employed to tailor porosity, mechanical strength, swelling behavior, and degradation kinetics. Contemporary hydrogels can be functionalized with bioactive agents—including growth factors, cytokines, antimicrobial peptides, stem cells, and extracellular vesicles—to regulate cell migration, enhance re-epithelialization, promote ECM remodelling, and modulate immune responses [7, 8].

Further advances have been achieved through integration of nanotechnology and stimuli-responsive design. Incorporation of inorganic nanoparticles (e.g., AgNPs, ZnO, and CeO₂) or polymeric nanocarriers enables antimicrobial activity and controlled drug delivery, while responsiveness to pH, temperature, redox state, or enzymatic activity allows hydrogels to dynamically adapt to pathological wound conditions. In addition, oxidative stress—a hallmark of chronic wounds—can be mitigated through ROS-scavenging nanoparticles, antioxidant small molecules, or enzymatic mimetics embedded within the hydrogel matrix, thereby protecting resident cells and supporting angiogenesis and tissue regeneration [8–10].

Among advanced wound-care biomaterials, injectable hydrogels have emerged as particularly attractive due to their minimally invasive administration, in situ gelation, and ability to conform to irregular wound geometries. Delivered as flowable precursors, these systems solidify in response to physiological triggers, such as pH or temperature, forming viscoelastic matrices that support cell infiltration, neovascularization, and tissue integration while reducing infection risk [11]. Importantly, injectable hydrogels can be engineered to exert immunomodulatory effects by regulating macrophage polarization, suppressing pro-inflammatory cytokines, and promoting pro-regenerative signaling pathways. Strategies such as PEGylation, zwitterionic surface modification, incorporation of natural polymers, and macrophage-modulating agents have further improved biocompatibility and reduced long-term immunogenicity, supported by ISO 10993-guided preclinical evaluations [12–14]. Recent innovations, including reversible and dynamic covalent crosslinking, have enhanced the mechanical adaptability, durability, and functional longevity of injectable hydrogels in complex wound environments [15]. Accumulating preclinical and early clinical evidence supports their potential to accelerate wound closure, enhance tissue regeneration, and reduce infection in chronic and non-healing wounds [16, 17].

The current review presents a comprehensive evaluation of injectable hydrogels in wound healing, emphasizing their physicochemical properties, biological mechanisms, and therapeutic implications. The discussion is framed to inform both researchers and clinicians about the latest advancements and future directions in hydrogel-based wound management.

### Definition of wound and wound healing process

A wound is defined as a disruption in the structural integrity of biological tissues, including the skin, mucous membranes, or organ tissues, resulting from a variety of traumatic agents. Effective management of wounds is critical to minimize the risk of infection and prevent further tissue damage [18]. Wound healing is a highly orchestrated biological process aimed at restoring tissue integrity, typically progressing through four distinct, interdependent phases: hemostasis, inflammation, proliferation, and remodelling. Each phase is marked by particular cellular and molecular events that collectively restore tissue homeostasis [19, 20].

Hemostasis, the first phase of wound healing, involves the activation of coagulation pathways, platelet aggregation, vasoconstriction, and localized glycolysis, which work to prevent excessive bleeding and supply a scaffold for the subsequent reparative processes [20, 21]. Subsequent to hemostasis, the inflammatory phase is defined by the attraction of immune cells, such as neutrophils and macrophages to the damaged area. These cells perform essential functions, including microbial clearance and the production of pro-inflammatory cytokines, which help regulate the healing process. However, persistent or excessive inflammation can impede healing by disrupting the balance between tissue destruction and repair, often resulting in chronic wounds [22]. The proliferative phase is marked by cellular events that promote tissue regeneration. Keratinocytes and fibroblasts migrate to the injury site, proliferate, and produce extracellular matrix (ECM) components necessary for re-epithelialization and angiogenesis [23]. The development of new vasculature is crucial to providing oxygen and essential nutrients to regenerating tissues. In the final phase, remodelling, collagen deposition and ECM reorganization occur, leading to the formation of mature, avascular, and acellular scar tissue. Fibroblasts undergo differentiation into myofibroblasts, which produce α-smooth muscle actin (SMA) and promote wound contraction, a process that is crucial for proper tissue closure [24].

Interruptions in any of these phases can result in prolonged healing times and the formation of chronic wounds. In addition, systemic conditions can impair angiogenesis, inflammatory resolution, and collagen synthesis, complicating the healing process [25]. Therefore, a deep understanding of the molecular and cellular mechanisms regulating wound healing is necessary for developing advanced treatment strategies aimed at promoting efficient recovery. The design of wound dressings must take into account both local and systemic factors that influence healing. Dressings should ideally promote cellular activities that facilitate wound closure while preventing infection and mitigating inflammation. Therefore, ongoing research is essential to develop wound care products that specifically address the complex pathophysiology of chronic wounds and improve patient outcomes [26, 27].

### Hydrogel efficacy in the wound healing process

Hydrogels have become integral to modern wound management due to their unique physicochemical and biological properties. These materials consist of three-dimensional networks of hydrophilic polymers capable of absorbing large quantities of water—often up to 1000 times their dry weight—depending on polymer composition and environmental conditions [28]. This high water content enables hydrogels to maintain a moist wound environment, a critical factor known to accelerate epithelialization, enhance autolytic debridement, and reduce pain. A key advantage of hydrogels is their ability to mimic the extracellular matrix (ECM), providing structural support and a hydrated, porous microenvironment that facilitates cellular migration, proliferation, and matrix deposition during tissue regeneration [29, 30]. Their biomimetic nature also enhances biocompatibility and minimizes immune responses, while biodegradable formulations ensure safe and predictable degradation without the need for removal [31]. Hydrogels can be engineered as bioadhesives, distributing mechanical loads uniformly and reducing localized stress at wound edges. This approach offers notable benefits over traditional closure techniques, lowering the risk of wound dehiscence and minimizing scar formation [32]. Their versatility further extends to controlled drug delivery: hydrogels can be functionalized with antimicrobial agents, growth factors, or anti-inflammatory molecules to modulate the wound microenvironment and prevent infection. Incorporating agents such as silver nanoparticles, essential oils, or antibiotics enables effective inhibition of pathogenic bacteria and subsequent reduction in inflammation [33]. In situ-forming hydrogels—formulated as low-viscosity pre-gel solutions—can rapidly undergo sol–gel transition upon exposure to physiological stimuli (e.g., pH, ionic strength, and temperature) [34].

### Difference between injectable hydrogels and conventional hydrogels

Conventional hydrogels are preformed three-dimensional polymer networks formed through physical or chemical crosslinking, yielding hydrated and porous structures that mimic key aspects of the extracellular matrix. Owing to their biocompatibility, permeability, and tunable mechanical properties, these materials have been widely used in drug and cell delivery, tissue engineering scaffolds, and medical devices [35, 36]. However, their clinical translation is often limited by implantation-related constraints. Preformed hydrogels typically require surgical placement, which increases procedural complexity, infection risk, patient discomfort, and recovery time [37]. In addition, their fixed geometry restricts conformal filling of irregular wound cavities or tissue defects, potentially compromising integration and therapeutic performance [38, 39].

Injectable hydrogels are designed to overcome these limitations through delivery as flowable pre-gel formulations that can be administered via standard syringes. Following injection, these systems undergo in situ gelation and conform to the geometry of complex defects, improving physical integration with surrounding tissues and enabling localized therapy [40]. Their formulation allows precise modulation of biochemical composition, mechanical strength, and degradation behavior to meet specific therapeutic requirements, including controlled drug release, tissue regeneration, and cell encapsulation [41, 42]. Many injectable hydrogels exhibit shear-thinning behavior, facilitating injection under mechanical stress while rapidly recovering network integrity after placement. In some systems, self-healing interactions further enhance mechanical stability under physiological deformation. The sol–gel transition is commonly triggered by physiological stimuli, such as temperature or pH, ensuring rapid stabilization and retention at the target site [43]. For clinical application, injectable hydrogels must also satisfy stringent physicochemical and biological criteria, including low pre-gel viscosity, cytocompatibility, and immunological inertness [44]. Although injectable hydrogels share fundamental characteristics with conventional hydrogels, they differ markedly in formulation strategy, mode of administration, and functional adaptability in vivo [45]. These distinctions underpin their increasing adoption in contemporary therapeutic approaches that require patient-specific defect filling (Table 1) [46].

**Table 1:** Summary of differences between injectable hydrogels and conventional hydrogels

Feature	Injectable hydrogels	Conventional hydrogels
Physical State	Low-viscosity liquid that gels upon injection	Typically pre-formed solid or semi-solid gel
Gelation Mechanism	In situ gelation triggered by physiological conditions	Often pre-crosslinked chemically or physically
Formulation Focus	Optimized for ease of injection and rapid gelation	May take various forms, not necessarily injectable
Applications	Primarily used in minimally invasive medical interventions	Broad range of applications across multiple fields
Viscosity	Low viscosity at room or physiological temperature	Varies widely depending on the composition
Biocompatibility	High, specifically designed for in vivo compatibility	Generally high, depending on materials and additives

### Natural and synthetic polymers for injectable hydrogels

Injectable hydrogels are extensively used in biomedical applications, including localized delivery of growth factors, chemotherapeutic agents, and antibiotics [47]. These hydrogels are synthesized from natural as well as synthetic polymers, with each providing distinct advantages. Natural polymers—derived from animals, plants, and microbial sources—include proteins, polysaccharides, and nucleic acids. Among them, plant-based polysaccharides are particularly valued for their biodegradability, biocompatibility, and inherent biological activities, such as antioxidant and anticoagulant properties, which can modulate immune responses [48].

### Natural polymers

Natural polymer-based injectable hydrogels, particularly those formulated from gelatin, chitosan, alginic acid, and hyaluronic acid, have demonstrated substantial promise in wound healing applications. These hydrogels can be synthesized in situ through various mechanisms, including Schiff-base reactions, enzyme-mediated crosslinking, photo-crosslinking, and temperature-responsive gelation [49]. Chitosan, a deacetylated derivative of chitin, is outstanding for its notable biocompatibility, biodegradability, and hemostatic properties [50]. Chitosan-based injectable hydrogels significantly promote wound healing by accelerating tissue regeneration and supporting drug delivery. Microspheres and nanoparticles derived from chitosan serve as effective carriers for the sustained release of therapeutic agents, particularly anti-inflammatory drugs [51]. In addition, chitosan has been explored as a safe and efficient vehicle for gene delivery applications due to its cationic nature, which enables it to form complexes with nucleic acids [8]. Alginic acid, a naturally derived anionic polysaccharide obtained from brown seaweed, is valued for its hydrophilicity, gel-forming ability, and favorable biological interactions [52]. Alginic acid-based hydrogels form stable, porous structures that facilitate cell adhesion and enhance wound repair. Hyaluronic acid (HA), a glycosaminoglycan found abundantly in the extracellular matrix, plays a vital role in tissue hydration, angiogenesis, and cell migration [53]. Injectable hydrogels containing HA have demonstrated effectiveness in wound healing by promoting fibroblast infiltration, enhancing re-epithelialization, and providing excellent mechanical strength [54, 55]. Gelatin, a transformed type of collagen, is frequently employed for its potential to facilitate cell adhesion, differentiation, and proliferation. Gelatin-based hydrogels have demonstrated positive outcomes in wound healing by mimicking the natural ECM and promoting tissue regeneration [56]. When mixed with other natural polymers, including konjac glucomannan, gelatin exhibits synergistic effects. For example, gelatin–chitosan hydrogels containing konjac glucomannan have been found to suppress inflammation and enhance wound closure in animal models [57]. In addition, β-chitin—a structural polysaccharide derived from marine organisms—has been chemically modified and incorporated into injectable hydrogel systems to further enhance wound healing efficacy [58]. Although protein-based hydrogels are less frequently used than polysaccharide-based ones due to longer gelation times and lower mechanical strength, they offer better biomimicry of natural ECM environments. This makes them advantageous for uses, including cell encapsulation and tissue engineering [59]. These hydrogels are often synthesized via free radical polymerization, wherein vinyl monomers are grafted onto protein backbones using crosslinking agents and initiators [60].

### Synthetic polymers

Synthetic polymers are pivotal in the development of injectable hydrogels due to their tunable properties, improved mechanical strength, and enhanced stability under physiological conditions. Unlike many natural polymers, which tend to degrade rapidly and exhibit weak mechanical behavior, synthetic copolymers often display more desirable characteristics for wound management applications. A defining feature of several synthetic polymers is their inverse sol–gel transition, wherein they undergo gelation upon heating rather than cooling [61]. Among the most commonly used are poly(ethylene oxide) (PEO) and poly(propylene oxide) (PPO), which form triblock copolymers known commercially as Pluronics. These polymers are water-soluble, and upon increasing temperature, PPO becomes less soluble, leading to enhanced hydrophobic interactions and the formation of spherical micelles. These micelles comprise a hydrophobic PPO core and a hydrophilic PEO corona [62, 63]. Due to their injectability, biocompatibility, safety, and thermo-reversible gelation at physiological temperatures, Pluronics are widely utilized in biomedical fields [64]. For example, Dang et al. developed injectable nanohydrogels based on synthetic polymers for burn wound therapy, demonstrating promising therapeutic results [65]. Poly(ethylene glycol) (PEG) and poly(lactic-co-glycolic acid) (PLGA) are two widely studied copolymers used in hydrogel synthesis. At lower temperatures, intermolecular hydrogen interactions among hydrophilic PEG segments facilitate a free-flowing state. Upon heating, the disruption of hydrogen bonds and the rise of hydrophobic interactions lead to gelation [65]. PLGA–PEG hydrogels exhibit advantageous features, such as biocompatibility, mechanical stability, biodegradability, and safety, making them suitable as scaffolds for cell growth and as drug delivery platforms [66]. For instance, teicoplanin-loaded PLGA–PEG–PLGA hydrogels were applied in a full-thickness excision wound model, significantly enhancing collagen deposition, angiogenesis, and wound closure while reducing inflammation [67]. Similarly, Lee et al. reported effective wound healing in diabetic models using PLGA–PEG–PLGA formulations [68].

Another prominent thermoresponsive polymer is poly(N-isopropylacrylamide) (PNIPAM), which displays a lower critical solution temperature (LCST), leading to a change from hydrophilic to hydrophobic properties, enabling gel formation. PNIPAM’s temperature sensitivity, crosslinking compatibility, biocompatibility, and deformability make it well-suited for wound-healing applications [69, 70]. A notable study employed free-radical polymerization to produce PNIPAM–cellulose nanocrystal (CNC) hybrid hydrogels, enhancing mechanical strength and thermal stability without additional crosslinkers [71]. PNIPAM-based hydrogels have consistently shown promising results in wound treatment by supporting sustained drug release and tissue regeneration [72, 73]. In addition to traditional synthetic polymers, self-assembling peptide-based hydrogels have emerged as innovative systems. These hydrogels rely on non-covalent interactions (e.g., electrostatic forces, hydrogen bonding, and π–π stacking), and are responsive to environmental stimuli, such as pH, temperature, enzymes, ionic strength, and ultrasound [74]. Owing to their non-toxicity, immunological inertness, biodegradability, and responsiveness to physiological cues, self-assembling peptide hydrogels offer a promising alternative for tissue engineering and drug delivery [11]. One study employed substance P-incorporated peptide hydrogels in diabetic wound models, which effectively stimulated collagen synthesis and angiogenesis [75]. Another study demonstrated that multi-domain peptide hydrogels promoted fibroblast proliferation and dermal regeneration in full-thickness cutaneous wound models in diabetic mice, corroborated by molecular assays [76].

## Methods of crosslinking

Hydrogels, as three-dimensional hydrophilic polymeric networks, function as artificial extracellular matrices (ECMs) by retaining large volumes of water while preserving their structural integrity. This unique ability stems from crosslinking between polymer chains, which prevents their dissolution or decomposition. Regardless of the polymer origin—natural or synthetic—two principal crosslinking strategies are employed in hydrogel formation: physical and chemical crosslinking [77].

### Physical crosslinking

**Fig. 1:**
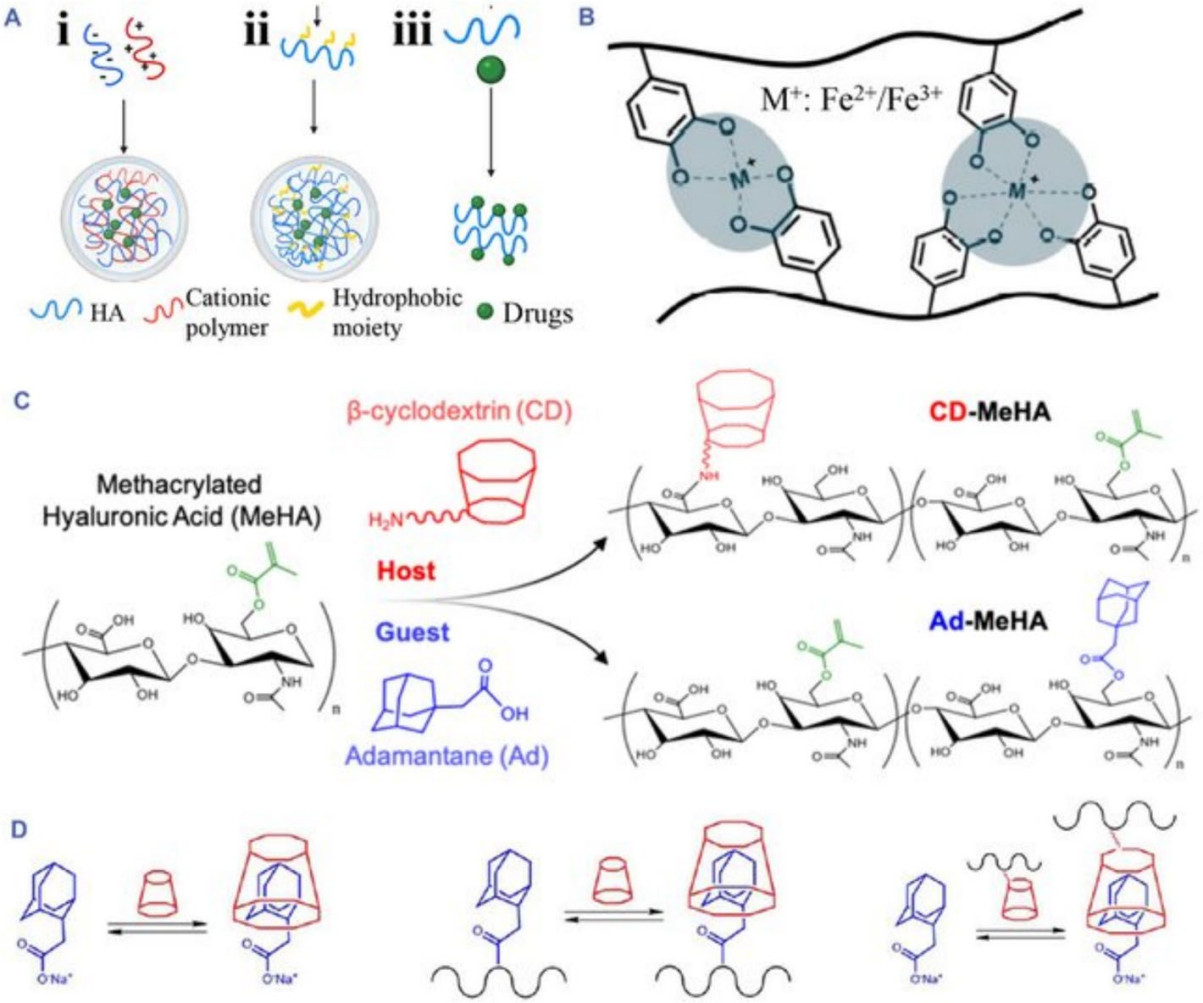
Physical crosslinking methods in hydrogels. **A** Schematic representation of physical crosslinking mechanisms: (i) electrostatic interaction, (ii) hydrophobic self-assembly, and (iii) hydrophobic/hydrophilic interaction. **B** Metal coordination of hyaluronic acid (HA) and calcium (ca) for crosslinking. Reproduced under the CC BY license. Copyright 2022, the authors, published by John Wiley and Sons [85]

Physical crosslinking encompasses various non-covalent interactions, such as ionic interactions, hydrogen bonding, amphiphilic self-assembly, freeze–thaw cycles, and protein-based interactions, that offer diverse strategies for hydrogel formation (Fig. 1) [78].

#### Ionic interactions

One of the most widely used physical crosslinking methods, ionic crosslinking, occurs rapidly under mild physiological conditions. For instance, alginate, a naturally sourced anionic polysaccharide composed of mannuronic and guluronic acid units, undergoes gelation in an environment containing divalent cations (e.g., Ca^2^⁺), forming an ionically crosslinked network. This approach allows for the quick and mild formation of hydrogels suitable for cell encapsulation and injectable therapies [78, 79].

#### Hydrogen bonding

Hydrogen bonds, formed between electron-deficient hydrogen atoms and electron-donating groups, contribute to the stability of physically crosslinked hydrogels. Although a single hydrogen bond is weak, networks formed through multivalent hydrogen bonding can exhibit sufficient mechanical integrity. These reversible interactions also lend injectable hydrogels self-healing and stimuli-responsive capabilities, important for dynamic biological environments [80].

#### Self-assembly of amphiphilic polymers

Amphiphilic block and graft copolymers can self-assemble in aqueous environments into well-defined structures characterized by hydrophobic cores and hydrophilic coronas. This self-organization leads to the formation of micelles or vesicles, which serve as physically crosslinked hydrogel networks. These systems are highly adaptable and can be engineered for targeted delivery and controlled release of bioactive agents [81, 82].

#### Freeze–thaw cycling

The freeze–thaw technique induces the formation of microcrystalline domains within polymer solutions, particularly in homopolymer systems, such as polyvinyl alcohol (PVA). This method enhances the mechanical strength and elasticity of hydrogels without requiring chemical agents. The reversible crystallization formed during repeated cycles of freezing and thawing acts as a physical crosslinking mechanism, producing hydrogels suitable for load-bearing or injectable applications [83].

#### Protein-based interactions

Protein interactions represent a sophisticated physical crosslinking approach. These involve genetically engineered proteins, peptide–peptide, or antigen–antibody interactions to establish interpenetrating polymer networks (IPNs). The tunability of protein-based hydrogels lies in the precision of genetic engineering, which allows researchers to design peptides with desired physicochemical properties. This control enables the development of hydrogels with precise degradation rates, mechanical behavior, and biological interactions [77, 84]. As research in biomaterials advances, novel physical crosslinking strategies continue to emerge, enabling fine-tuned, stimuli-responsive, and cell-friendly injectable hydrogels that mimic native ECMs. These methods form the foundation for future innovations in tissue engineering, wound repair, and targeted drug delivery.

### Chemical crosslinking

**Fig. 2:**
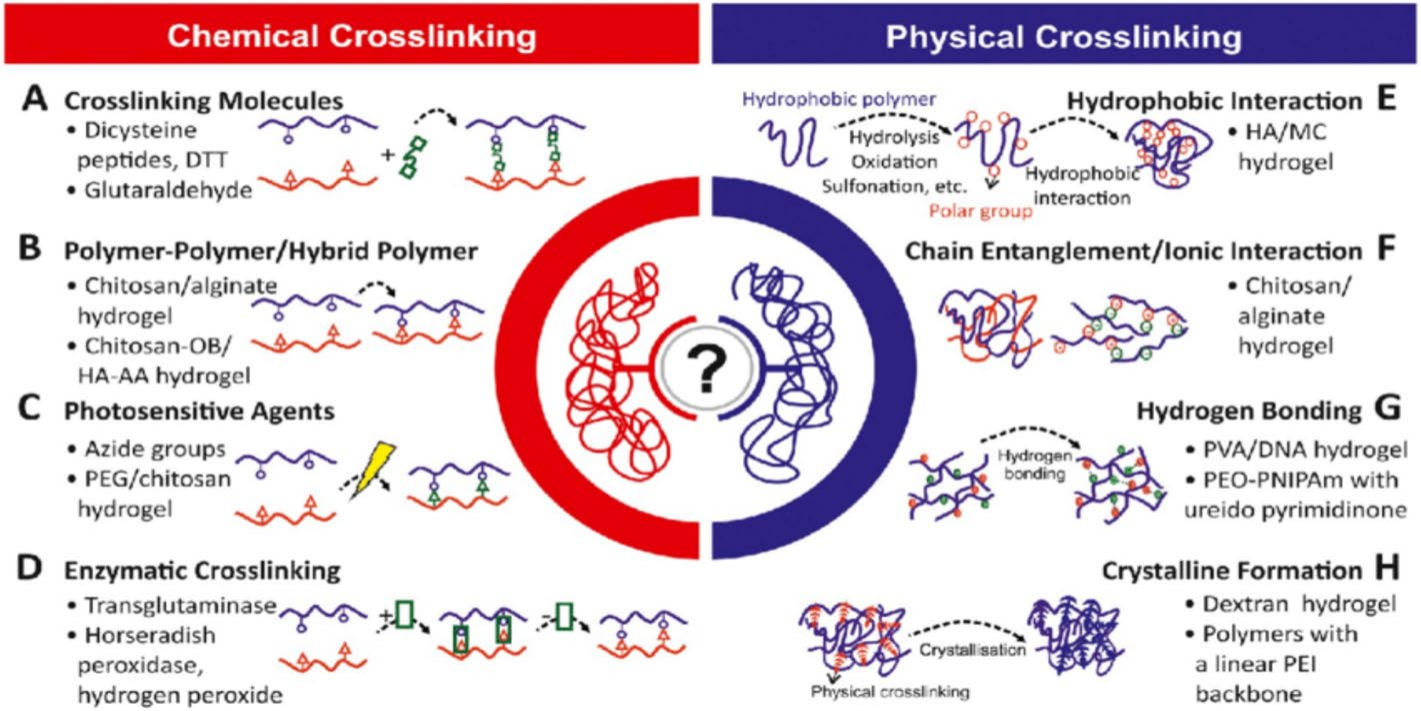
Strategies for hydrogel synthesis. Examples of chemical and physical crosslinking approaches are presented [90]

Chemical crosslinking is an extensively used technique for creating hydrogels, providing superior strength and durability compared to physical crosslinking methods (Fig. 2). This approach utilizes bifunctional crosslinking agents to form covalent linkages between polymer chains, leading to stable and robust hydrogel structures. Chemical crosslinking includes several processes, such as chemical reactions, enzymatic reactions, high-energy irradiation, and free radical polymerization, all of which contribute to improve the mechanical properties of hydrogels [86]. In chemical reactions, covalent bonds form between polymer chains through the reactivity of functional groups. For instance, isocyanate groups may react with hydroxyl (OH), amine (NH₂), or carboxylic acid groups to facilitate the formation of Schiff bases, which are critical for hydrogel formation [87]. Enzymatic crosslinking utilizes enzymes to generate covalent bonds between polymer chains modified with enzyme-sensitive compounds. Several enzymes, such as tyrosinase, transglutaminase, lysyl oxidase, phosphatases, phosphopantetheine transferase, plasma amine oxidase, horseradish peroxidase mimetics, and peroxidases, can be employed in this process [88]. Free-radical polymerization and high-energy irradiation are methods used to generate free radicals, which initiate the formation of cross-linked polymer networks. These free radicals are produced through enzyme catalysts or UV light excitation, promoting the formation of irreversible covalent bonds and the development of a crosslinked hydrogel structure [89].

### Bio-orthogonal chemistries for next-generation injectable hydrogels

Bio-orthogonal chemistries provide a chemically selective framework for constructing injectable hydrogels in complex biological settings, where conventional crosslinking reactions often suffer from nonspecific side reactions or cytotoxic catalysts. By enabling reactions that proceed independently of native cellular components, bio-orthogonal strategies allow hydrogel formation and post-injection modification to occur without perturbing surrounding biological processes, making them particularly suitable for wound healing and regenerative medicine applications [91, 92]. These features establish bio-orthogonality as a foundational design principle rather than merely a crosslinking alternative. Among these strategies, next-generation click reactions—most notably strain-promoted azide–alkyne cycloaddition (SPAAC) and tetrazine–trans-cyclooctene (Tz–TCO) ligation—offer distinct kinetic and chemical advantages. Their rapid reaction rates and high chemoselectivity enable efficient network formation or functional group conjugation immediately following injection, even at low reactant concentrations, without the need for metal catalysts or external triggers [93, 94]. Ongoing molecular optimization efforts are focused on improving reaction kinetics under dilute conditions, minimizing steric limitations within dense polymer networks, and enhancing bond stability in aqueous environments to support clinical translation. Dynamic and reversible bio-orthogonal linkages represent an additional layer of functional sophistication. Chemistries based on oxime, hydrazone, boronate ester, and disulfide bonds introduce covalent adaptability into injectable hydrogel networks, allowing reversible bond exchange under defined chemical or mechanical stimuli [95, 96]. This dynamic behavior supports self-healing, stress relaxation, and programmable degradation, which are critical for maintaining hydrogel integrity in mechanically active and heterogeneous wound sites. Importantly, the reversibility of these linkages enables post-injection adjustment of network mechanics and drug release behavior without requiring material removal or replacement [97]. Photo-activated bio-orthogonal reactions further expand the functional design space of injectable hydrogels by introducing externally addressable control. Visible- and near-infrared-responsive photo-click reactions permit localized crosslinking, bond cleavage, or functional activation with high spatial resolution following material deployment [98]. The use of tissue-penetrating wavelengths enables non-contact modulation of hydrogel properties after injection, offering a means to dynamically adjust material performance in chronic or anatomically challenging wounds. Looking forward, orthogonal reaction networks that integrate multiple chemically independent bio-orthogonal transformations within a single hydrogel platform are expected to enable hierarchical and modular functionality. Such systems allow distinct therapeutic components—such as antimicrobial agents, growth factors, and immunomodulatory signals—to be introduced or released through independent reaction pathways in response to evolving biological cues [99]. When combined with microenvironment-responsive elements, including enzyme-, reactive oxygen species-, and pH-sensitive motifs, these platforms can autonomously regulate degradation, mechanics, and therapeutic output in synchrony with wound progression [103].

## Bioactive agents used for functionalizing

### Hemostasis

Hydrogels are three-dimensional networks created by the cross-linking of hydrophilic polymers, with holes capable of holding large volumes of water or aqueous solutions [100]. These hydrogels are used as hemostatic agents, because they support a natural cellular environment, adapt to soft and hard tissues, and have tunable physical, chemical, and biological properties [101]. Hemostatic hydrogels are distinguished by their quick gelation, high biocompatibility, superior mechanical characteristics, and adequate adhesion [102]. Alginate-based hydrogels have a long history of application in hemostasis. After absorbing blood and other body fluids, alginate hydrogels adhere to the wound surface, seal capillaries and microvessels, and provide physical pressure to the bleeding site, resulting in successful hemostasis [103]. Calcium alginate, a typical hemostatic substance, contains phytohemagglutinin, which can clump red blood cells and change their shape. This mechanism exposes erythrocyte-surface phosphatidylserine, allowing the local conversion of prothrombin to thrombin and, therefore, speeding hemostasis [104]. Gelatin is another synthetic substance used to maintain hemostasis. Gelatin-based hemostatic hydrogels can be strengthened with functional nanoparticles to improve their inherent characteristics and suitability for hemostatic applications [105]. For example, Choi et al. created an innovative mussel-inspired tissue adhesive hydrogel, DOPAFe3 + gelatin, and tested its adhesive characteristics and hemostatic efficacy in a rat model of haemorrhage liver. The study found that this hydrogel was effective at achieving hemostasis [106]. Keratin, an insoluble structural protein high in sulfur-containing cysteine amino acids, can be found in hair, nails, feathers, wool, and horn. Keratin has hemostatic properties that reduce blood clotting time, plasma clotting lag periods, and promote an ECM-like structure. It also serves as a catalytic surface for the clotting cascade and promotes the polymerization of fibrinogen into fibrin [107]. Numerous research have looked into the usage of keratin in wound healing and found excellent outcomes [108, 109]. Chitosan improves platelet function by raising the expression of GPIIb/IIIa (a platelet surface receptor) and mobilizing [Ca2 +], resulting in powerful platelet aggregation [110]. This action is attributable to chitosan's positively charged amino groups, which interact electrostatically with negatively charged groups present on erythrocyte membranes and proteins, resulting in significant hemagglutination at damaged regions [111]. Chitosan can also cause hemolysis, which leads to haemoglobin externalization [112]. Several investigations have shown that chitosan in injectable hydrogels improves hemostasis during wound healing [50, 113, 114].

### Antibacterial activity

#### Advances in the stimuli-responsive injectable hydrogels for controlled release of drugs

Stimuli-responsive injectable hydrogels are designed to translate wound-specific biochemical signals into controlled changes in network structure, degradation behavior, and therapeutic release profiles. In infected and chronic wounds, pathological cues, such as acidic pH, elevated protease activity, increased reactive oxygen species (ROS), and sustained inflammation provide exploitable triggers for selective hydrogel activation, whereas resolving wounds exhibit near-physiological conditions that favor material stability and prolonged release [115]. pH-responsive injectable hydrogels utilize ionizable moieties or acid–labile linkers to induce swelling, bond cleavage, or charge conversion under acidic conditions, enabling rapid or on-demand antimicrobial release specifically within infected regions while maintaining slower release kinetics at neutral pH. Precise tuning of pKa, crosslink density, and mesh size allows control over activation thresholds and release rates to match clinically relevant wound acidity profiles [116].

Enzyme-responsive systems further enhance spatial and temporal selectivity by incorporating peptide or polymeric sequences that are substrates for matrix metalloproteinases (MMPs), neutrophil elastase, or pathogen-derived proteases. Enzyme-cleavable crosslinks promote localized hydrogel degradation and cargo liberation in protease-rich environments, while remaining largely inert in healthy tissue, thereby reducing off-target exposure and supporting staged wound healing [117]. Dynamic covalent bonds (e.g., hydrazone, oxime, boronate ester, and disulfide linkages), along with noncovalent shear-responsive interactions, introduce viscoelasticity, stress relaxation, and self-healing behavior into injectable hydrogel networks. These adaptable properties enable dissipation of injection-induced shear, restoration of structural integrity in situ, and accommodation of tissue motion while allowing programmed degradation in response to microenvironmental cues. Such mechanical adaptability has been shown to facilitate cell infiltration, mitigate chronic inflammation, and better match the evolving mechanical demands of regenerating tissue [118]. More advanced platforms integrate orthogonal and multi-stimuli trigger systems**,** combining pH-, enzyme-, ROS-, or light-responsive elements within a single injectable hydrogel. This hierarchical design enables differential release profiles, such as rapid antimicrobial delivery in infected niches followed by sustained release of growth factors to support matrix remodeling and tissue regeneration. Cascade-triggered and orthogonal chemistries allow independent tuning of antimicrobial, immunomodulatory, and regenerative functions, which is particularly relevant for chronic wounds that progress through distinct healing phases [119].

#### Injectable hydrogels with intrinsic and engineered antibacterial functions

**Fig. 3:**
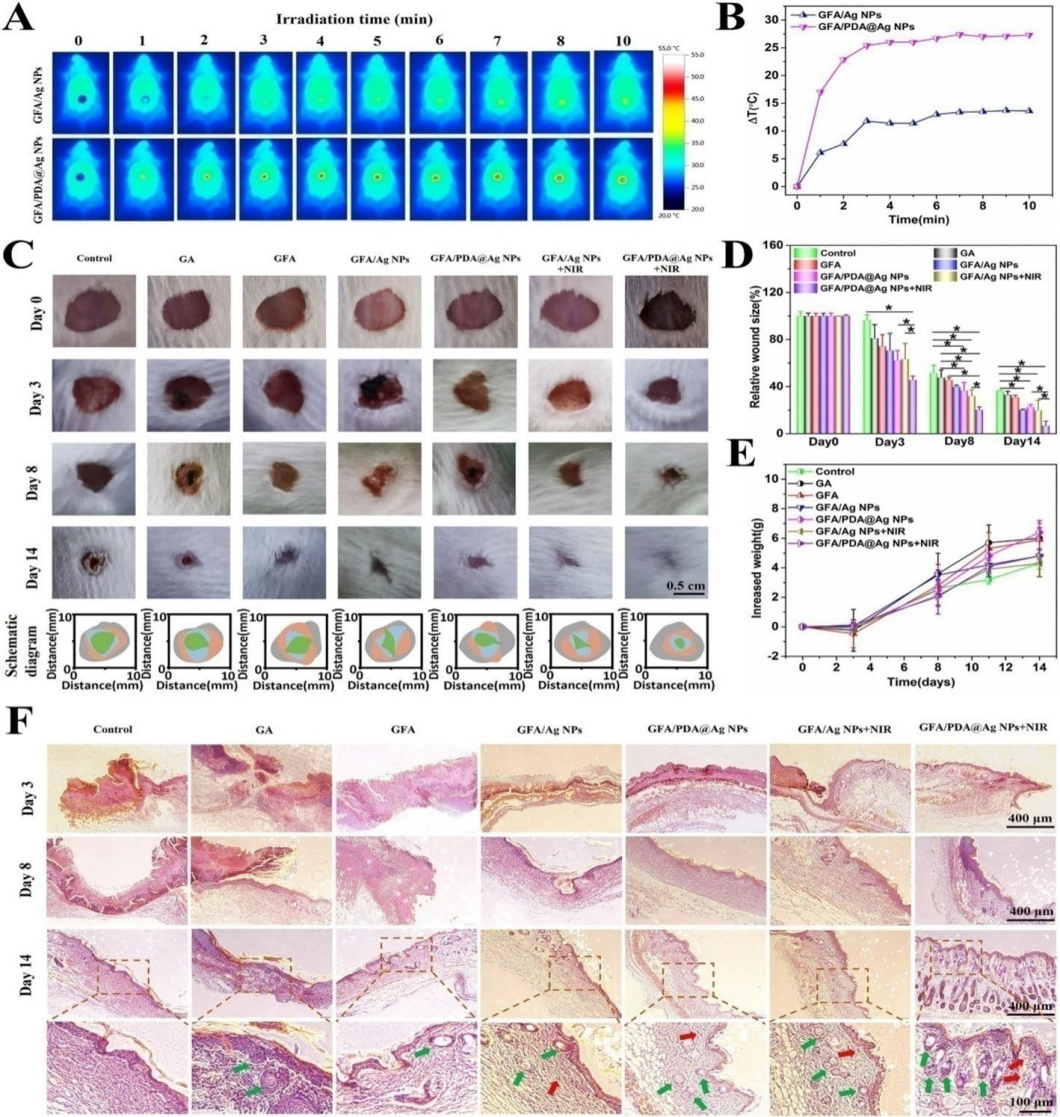
**A** Thermal imaging of GFA/Ag NPs hydrogel and GFA/PDA@Ag NPs hydrogel, both irradiated by NIR laser. *Reproduced with permission from Ref* [129]

Beyond stimuli-responsive release, injectable hydrogels can exert antibacterial activity through intrinsic material properties or engineered antimicrobial components. Chitosan-based hydrogels exhibit inherent antibacterial effects mediated by electrostatic interactions between the protonated amino groups of glucosamine and negatively charged bacterial membranes. These interactions disrupt membrane integrity, induce osmotic imbalance, interfere with intracellular processes, and ultimately lead to bacterial cell death [120]. Numerous studies have demonstrated the effectiveness of chitosan when incorporated into injectable hydrogel systems for infection control [7, 121, 122]. Nitric oxide (NO) represents another potent antibacterial agent, exerting broad-spectrum activity through oxidative and nitrosative stress pathways. Injectable hydrogels incorporating NO donors, such as S-nitrosothiolated gelatin, have shown effective bacterial eradication and infection prevention [123]. Antimicrobial peptides (AMPs) play a critical role in host defense, particularly against antibiotic-resistant pathogens. Their amphiphilic and polycationic structures facilitate interaction with negatively charged bacterial membranes, leading to membrane disruption, inhibition of nucleic acid and protein synthesis, and bacterial death [124]. Peptide-based injectable hydrogels, including β-hairpin scaffolds reported by Salick et al*.*, demonstrate contact-mediated bacterial membrane disruption and rapid bactericidal activity [125]. Similarly, arginine-rich peptide hydrogels exhibit strong interactions with phospholipid-rich bacterial membranes via electrostatic and hydrogen bonding interactions [126]. Nandi et al*.* further demonstrated that modulation of alkyl chain length in peptide-based hydrogels significantly influences antibacterial potency across multiple bacterial strains [127]. Recent advances highlight multifunctional injectable hydrogels that integrate intrinsic antibacterial activity with additional therapeutic modalities [128]. For example, hydrogels formed through dynamic covalent and coordination bonds—such as oxidized alginate combined with catechol-modified gelatin—have been loaded with polydopamine-coated silver nanoparticles (Fig. 3), enabling sustained antibacterial release and photothermal sterilization under near-infrared irradiation [129]. In vivo studies demonstrate that these systems effectively suppress inflammation, promote angiogenesis, and accelerate wound closure, underscoring the potential of injectable antibacterial hydrogels to simultaneously address infection control and tissue repair [130].

#### Antimicrobial injectable hydrogels and biofilm-associated challenges

Despite their antibacterial functionality, injectable hydrogels remain susceptible to biofilm formation, particularly during long-term application in chronic wound environments. Factors such as heterogeneous antimicrobial distribution, gradual depletion or inactivation of active agents, adsorption of host-derived proteins, and surface properties conducive to bacterial adhesion collectively promote microbial colonization at the hydrogel–tissue interface [131]. Once established, biofilms exhibit pronounced tolerance to antimicrobial therapies and host immune responses due to the protective extracellular polymeric matrix, reduced bacterial metabolic activity, and activation of stress-response pathways. Persistent biofilms sustain chronic inflammation, impair angiogenesis and re-epithelialization, and represent a major barrier to wound resolution, especially in diabetic and ischemic wounds [132, 133]. To overcome these limitations, next-generation antimicrobial injectable hydrogels increasingly incorporate multifunctional antibiofilm strategies**.** Contact-active antimicrobial surfaces—functionalized with cationic polymers, antimicrobial peptides, or quaternary ammonium groups—enable direct bacterial membrane disruption upon adhesion, reducing reliance on diffusion-based killing. In parallel, anti-fouling chemistries such as zwitterionic or highly hydrophilic polymer coatings suppress nonspecific protein adsorption and initial bacterial attachment, thereby inhibiting biofilm nucleation [134].

## Industrial scale-up of injectable hydrogels: regulatory frameworks and evaluation methodologies

The successful industrialization and clinical translation of injectable hydrogels require strict adherence to regulatory standards and comprehensive physicochemical, mechanical, and biological characterization. Aligning formulation, manufacturing, and quality-control processes with established guidelines—particularly those of the European Pharmacopoeia (Ph. Eur.), ISO, and ASTM—is essential to ensuring product safety, batch uniformity, and therapeutic performance. This section outlines the principal regulatory considerations and analytical methodologies used to validate injectable hydrogel systems for biomedical applications [39].

### Regulatory frameworks

The European Pharmacopoeia (Ph. Eur.) provides the primary regulatory foundation governing the quality and safety of injectable hydrogel-based biomedical products. Its monographs and technical specifications define critical quality attributes (CQAs) that must be met prior to clinical translation. These include rigorous assessment of identity and purity, ensuring accurate chemical composition and the elimination of residual monomers, unreacted crosslinkers, synthesis by-products, or other process-related impurities. Potency and performance parameters must be quantified to confirm consistent therapeutic activity and reproducible functional behavior across production batches. Stability profiling—typically involving controlled exposure to variations in temperature, relative humidity, and light—further establishes the hydrogel’s physicochemical robustness and capacity to maintain structural and biological integrity over its intended shelf life and during clinical application. In addition, stringent evaluation of sterility and pyrogenicity, including bacterial endotoxin quantification, is required to mitigate infection risks and prevent adverse inflammatory or febrile responses following administration.

Complementing the requirements of Ph. Eur., the ISO 10993 series provides globally recognized standards for biological risk assessment of medical materials. These guidelines encompass a comprehensive panel of tests evaluating cytotoxicity, sensitization, irritation or intracutaneous reactivity, acute and chronic systemic toxicity, hemocompatibility, and implantation responses. Collectively, ISO 10993 frameworks enable thorough characterization of the biocompatibility profile, biodegradation behavior, and long-term host–material interactions of injectable hydrogels intended for wound healing and regenerative medicine applications. Together, Ph. Eur. and ISO standards establish a robust regulatory and analytical foundation essential for ensuring the safety, efficacy, and clinical reliability of next-generation injectable hydrogel therapies [39].

### Thermomechanical evaluation

Thermal and mechanical evaluations are fundamental for determining the suitability of injectable hydrogels in biomedical applications, particularly wound healing. These assessments provide essential information on rigidity, strength, viscoelasticity, and thermal stability under physiologically relevant conditions [135]. From a mechanical standpoint, characterization ensures that hydrogels can endure physiological stresses without compromising structural integrity or functional performance. Similarly, thermal stability is critical for storage, handling, and reliable in vivo application. Collectively, thermomechanical data guide the selection of polymers and optimization of crosslinking strategies to meet specific clinical requirements [136]. Mechanical assessments quantify rigidity—defined as resistance to deformation—and strength, the maximum stress tolerated before material failure. Tensile and compression tests are commonly used to determine the Young’s modulus, which reflects the elastic behavior of hydrogels [137]. Additional insight into viscoelasticity and durability is obtained through stress–strain analyses and dynamic mechanical analysis (DMA) performed under dynamic loading conditions [138]. Evaluating cyclic loading resistance is particularly important for hydrogels intended for soft tissues that experience repeated deformation during normal physiological activity [139, 140]. Thermal stability is typically examined using differential scanning calorimetry (DSC), which identifies thermal transitions, such as melting (Tm) and glass transition (Tg). Thermogravimetric analysis (TGA) provides information on degradation profiles and moisture content, contributing to the development of appropriate storage and sterilization protocols. Furthermore, temperature- and frequency-dependent DMA elucidates thermomechanical stability, a key predictor of in vivo performance [135].

### Microstructural evaluation

Microstructural and morphological analyses play a central role in optimizing hydrogel function and performance. Microscopic techniques—including SEM, TEM, STM, AFM, and confocal microscopy—provide detailed visualization of surface characteristics, pore architecture, and the internal polymeric network. More advanced methods, such as environmental SEM (ESEM), Cryo-SEM, micro-computed tomography (µCT), and second-harmonic generation (SHG) imaging enable three-dimensional assessment of hydrogel architecture and hydration states [141]. Structural parameters such as pore size, porosity, and surface morphology directly influence nutrient transport, cellular infiltration, and tissue integration. Comprehensive evaluation of structural integrity, mechanical robustness, and distribution of composite components ensures reproducibility, quality control, and long-term clinical reliability [142, 143].

### Bioevaluation and biocompatibility assessment

Injectable hydrogels commonly employ natural polymers—including hyaluronic acid, chitosan, alginate, and heparin—owing to their excellent biocompatibility and low immunogenicity [144]. These biomaterials inherently support cellular adhesion, proliferation, and differentiation, and in some cases exhibit intrinsic therapeutic functions, such as angiogenesis promotion and anti-inflammatory activity [145].

Biological evaluation encompasses both in vitro and in vivo assessments. In vitro cytotoxicity assays identify potential cellular toxicity, while in vivo biocompatibility studies assess tissue responses, inflammatory reactions, and overall safety following implantation. In addition, drug-release studies characterize the release kinetics of incorporated therapeutics, providing essential information for the development of controlled or sustained drug-delivery systems [146–148].

### Physical and chemical evaluation

The physical and chemical characteristics of hydrogels—governed by functional groups, crosslinking density, and intermolecular interactions—directly affect their performance across biomedical applications. Stability and degradation analyses evaluate how hydrogel properties evolve under various environmental influences, such as temperature, pH, moisture, solvents, enzymatic activity, and radiation exposure, all of which may alter network integrity or chemical functionality [39, 149]. Swelling behavior, quantified by immersing lyophilized samples in physiological media and measuring water uptake, is a critical parameter influencing drug-loading capacity and release kinetics. Higher swelling ratios often correlate with improved therapeutic loading and sustained release capabilities [150, 151]. Gelation time, which represents the transition from liquid to semi-solid following mixing or injection, is another key parameter for in situ-forming hydrogels. Typically assessed using the inverted vial method, gelation kinetics are affected by molecular weight, polymer concentration, phenolic content, and solubility—all of which influence crosslinking behavior [39, 152–154]. Rheological properties, including viscosity, shear-thinning behavior, and elasticity, dictate the ease of injection and mechanical performance following administration. These characteristics are commonly evaluated using oscillatory shear rheology and rotational viscometry. Rheological stability is strongly influenced by molecular weight, crosslinking density, and polymer–solvent interactions [39, 155, 156]. Syringeability and injectability refer to the force required to expel hydrogels through a syringe, parameters directly related to viscosity and rheological behavior. Optimizing these properties is essential to ensure clinical usability, procedural efficiency, and patient comfort [157, 158].

#### *Defining and standardizing optimal gelation time for *in situ* injectable hydrogels*

The definition of an “optimal” gelation time for in situ-forming injectable hydrogels is inherently multifactorial, requiring careful reconciliation of clinical handling requirements, physicochemical performance, and biological compatibility. Rather than representing a single fixed value, optimal gelation time is more appropriately conceptualized as a functional temporal window that ensures injectability, precise spatial retention at the target site, and safe interaction with surrounding tissues [159, 160]. From a clinical handling perspective, gelation must proceed sufficiently slowly to allow smooth syringe extrusion, homogeneous mixing of precursor components, and conformal filling of irregular wound or tissue geometries while occurring rapidly enough to prevent material leakage, dilution by wound exudate, or displacement from the application site [161, 162]. In wound healing and soft tissue applications, gelation times spanning several seconds to a few minutes are generally considered acceptable, depending on anatomical location and delivery route. Excessively rapid gelation may result in premature solidification, needle clogging, and incomplete defect filling, whereas overly delayed gelation increases the risk of loss of localization and reduced therapeutic efficacy [163].

From a materials characterization standpoint, gelation time is most rigorously defined using time-resolved oscillatory rheology, where the sol–gel transition is identified by the crossover point at which the storage modulus (G′) exceeds the loss modulus (G″), signifying the formation of a percolated elastic network. This rheological criterion provides a quantitative, reproducible metric under controlled experimental conditions and is widely adopted in injectable hydrogel research. Complementary qualitative methods, such as vial inversion tests, are frequently reported for practical comparison; however, these approaches are inherently operator-dependent and lack methodological standardization [164, 165]. Biological considerations further refine the definition of optimal gelation time. In situ gelation must occur under physiological temperature, pH, and ionic strength, without generating cytotoxic intermediates, excessive heat, or abrupt mechanical stresses. Importantly, gelation kinetics should be compatible with cell viability, protein stability, and bioactive molecule retention, particularly in applications involving cell encapsulation or localized delivery of growth factors and enzymes. Rapid crosslinking has been shown to impair cell spreading and mechanotransduction, whereas delayed gelation may compromise spatial control and encapsulation efficiency [166, 167]. Despite its critical importance, full standardization of gelation time remains challenging due to substantial variability in polymer chemistry, crosslinking mechanisms (physical versus covalent), precursor concentration, and testing methodologies. Current best practices emphasize reporting gelation kinetics under well-defined experimental conditions—including temperature, shear history, polymer concentration, and measurement technique—to enable meaningful comparison across studies [168, 169]. While international guidelines such as ASTM F2450 provide frameworks for evaluating injectability and handling of biomaterials, and ISO 10993 outlines biological safety requirements, no universal standard currently prescribes a single optimal gelation time applicable to all injectable hydrogel systems [170, 171].

### Analytical techniques for chemical and structural assurance

Spectroscopic and spectrometric analyses provide essential chemical and structural verification of injectable hydrogels. NMR elucidates polymer backbone structure and molecular dynamics, while FTIR identifies functional groups and confirms chemical modifications. UV–Vis spectroscopy assesses chromophores and drug-loading profiles. Raman spectroscopy offers complementary vibrational data related to molecular interactions and crosslinking. Mass spectrometry supports molecular weight determination and detects residual monomers or degradation products. Collectively, these methods ensure regulatory-grade chemical characterization and material integrity [172–174].

By integrating comprehensive mechanical, thermal, microstructural, and spectroscopic analyses, researchers can establish rigorous quality benchmarks that ensure injectable hydrogels satisfy the safety, efficacy, and performance criteria required for clinical translation and advanced applications in tissue engineering and regenerative medicine. Continued advancements in characterization methodologies, coupled with adherence to regulatory standards, remain essential for facilitating the successful transition of injectable hydrogel technologies from the laboratory to clinical practice.

## Translational barriers of injectable hydrogels

### Physicochemical and manufacturing variability

Hydrogel systems frequently exhibit pronounced batch-to-batch variability in network formation, crosslinking density, and structural organization, which directly impacts reproducibility, mechanical performance, and therapeutic payload release profiles in vivo. Such variability poses a substantial barrier to clinical translation, as consistent physicochemical properties are essential for meeting regulatory expectations and ensuring predictable clinical outcomes [175]. Manufacturing-related heterogeneity may arise from fluctuations in polymer molecular weight, precursor concentration, crosslinking kinetics, and environmental conditions during synthesis, all of which can alter gelation behavior, injectability, and degradation dynamics [40].

Achieving robust control over these parameters during scale-up remains particularly challenging, as laboratory-scale fabrication methods often fail to translate directly to Good Manufacturing Practice (GMP)-compliant production. This disconnect complicates process validation and limits the reproducibility required for regulatory approval and clinical reliability [176].

Emerging manufacturing strategies, including microfluidic-assisted synthesis, automated mixing systems, and tightly standardized crosslinking protocols, offer promising avenues to enhance structural uniformity and process reproducibility [177, 178]. In parallel, implementation of in-line process analytical technologies (PAT) and rigorous quality-by-design (QbD) frameworks is increasingly recognized as critical for defining acceptable variability ranges and ensuring batch consistency [179, 180]. Addressing physicochemical and manufacturing variability through such integrated approaches is essential for advancing hydrogels from experimental platforms toward scalable, clinically viable therapeutic systems.

### Safety, biodistribution, and long-term biocompatibility

The clinical translation of injectable hydrogel systems critically depends on their long-term safety, in vivo fate, and biocompatibility following administration. Although injectable hydrogels are designed to provide localized therapy, their degradation products, crosslinking agents, and encapsulated bioactive components inevitably interact with surrounding tissues and systemic circulation, raising important considerations regarding biodistribution, immunogenicity, and long-term tissue responses [181]. In vivo stability and degradation kinetics play a central role in determining hydrogel residence time and clearance pathways [182]. Hydrogels that degrade too slowly may persist beyond the therapeutic window, potentially leading to chronic foreign-body responses, fibrotic encapsulation, or sustained low-grade inflammation. Conversely, overly rapid degradation can result in premature loss of mechanical integrity, uncontrolled release of payloads, and systemic exposure to polymer fragments. The physicochemical properties of the hydrogel network—including polymer composition, crosslinking chemistry, and mesh size—strongly influence degradation behavior and the biodistribution of soluble degradation products [183]. Biocompatibility concerns extend beyond acute cytotoxicity to include long-term immune interactions. Injectable hydrogels may modulate local immune cell recruitment and macrophage polarization, which can either support tissue regeneration or promote chronic inflammation, depending on material composition and degradation by-products. Residual crosslinkers, unreacted monomers, or reactive intermediates generated during in situ gelation can further exacerbate inflammatory responses and complicate safety profiles [184].

Repeated or high-volume administration introduces additional translational challenges, as cumulative exposure to hydrogel components and their degradation products may lead to systemic accumulation, altered organ function, or immune sensitization over time. These risks are particularly relevant for chronic wound management and regenerative therapies that require multiple injections or prolonged material residence [45].

Accordingly, comprehensive preclinical evaluation is essential and should encompass acute and chronic toxicity, local tissue response, degradation and clearance pathways, and immune compatibility under physiologically relevant conditions. Long-term in vivo studies, coupled with standardized histopathological, biochemical, and immunological assessments, are necessary to establish robust safety profiles. Alignment with international standards, including ISO 10993 for biological evaluation of medical devices, is critical to facilitate regulatory approval and successful clinical translation of injectable hydrogel technologies [185, 186].

### Controlled therapeutic loading and release

Achieving reproducible therapeutic loading and precise spatiotemporal release of bioactive agents—such as growth factors, antimicrobial drugs, or nucleic acid-based therapeutics—remains a central challenge in the clinical translation of injectable hydrogel systems. This challenge is particularly pronounced for fragile biologics and poorly water-soluble compounds, whose stability and bioactivity can be compromised during encapsulation, in situ gelation, or prolonged residence within hydrophilic polymer networks [187].

Injectable hydrogels often rely on aqueous precursor formulations, which may limit the loading efficiency of hydrophobic drugs and increase the risk of heterogeneous distribution within the gel matrix. Furthermore, weak physical interactions between the therapeutic agents and the polymer network frequently result in an initial burst release, followed by subtherapeutic release profiles that undermine treatment efficacy and duration. In the case of sensitive biomolecules, such as proteins and nucleic acids, exposure to shear forces during injection, changes in pH or ionic strength, and reactive crosslinking chemistries may further reduce biological activity [188].

### Regulatory and scale-up barriers

Injectable hydrogels face substantial regulatory and translational challenges owing to their hybrid nature, which often combines device-like structural functions with localized therapeutic activity. This dual functionality complicates regulatory classification, approval pathways, and standardization of evaluation criteria. Although frameworks such as ISO 10993 address biological safety, they do not fully capture the dynamic in situ behavior of injectable hydrogels, including gelation kinetics, degradation profiles, and long-term tissue interactions, necessitating extensive product-specific preclinical assessment [189].

Manufacturing scale-up represents an additional barrier, as clinical translation requires strict batch-to-batch consistency in polymer composition, crosslinking efficiency, sterility, and rheological performance. Early adoption of Good Manufacturing Practice (GMP)-compliant processes, validated analytical controls, and reproducible fabrication protocols is essential to ensure clinical reliability and regulatory acceptance. Failure to integrate regulatory strategy and manufacturability at early development stages remains a major contributor to translational attrition [190].

## Applications of injectable hydrogels in promoting wound healing

Injectable hydrogels have emerged as highly versatile wound-healing platforms due to their favorable physicochemical properties, capacity for in situ gelation, and ability to conform to irregular tissue geometries. Their injectable nature enables uniform coverage of deep or complex wounds while minimizing procedural trauma. For instance, a thermoreversible hyaluronic acid (HA)/κ-carrageenan hydrogel exhibited rapid in situ gelation and maintained a moist microenvironment favorable for tissue repair in a rodent model [191].

Beyond physical protection, injectable hydrogels function as active therapeutic depots capable of modulating the wound microenvironment. An HA–chitosan–glycerophosphate hydrogel loaded with ciprofloxacin and carvacrol demonstrated sustained antimicrobial release, reduced bacterial load, and significantly enhanced wound closure in a rabbit ischemic-wound model (~ 51.2% wound area by day 9 versus ~ 81.8% in controls) [192].

Their intrinsic hydrophilicity maintains wound hydration, facilitating autolytic debridement, granulation tissue formation, angiogenesis, and re-epithelialization while minimizing scar formation [31]. Material versatility enables injectable hydrogels to be tailored for diverse wound types—including acute, chronic, ischemic, and infected wounds. Chitosan-based hydrogels, for example, combine inherent hemostatic, antimicrobial, and bioadhesive behavior with excellent injectability and self-healing capacity, making them highly suitable for complex wound management [193]. Furthermore, multifunctional hydrogels incorporating antioxidant moieties, adhesive components, or dynamic crosslinking chemistries can simultaneously counteract oxidative stress, enhance tissue adhesion, and promote coordinated tissue regeneration. A gelatin/HA/collagen III/tannic acid hydrogel demonstrated strong cytocompatibility, robust ROS-scavenging (~ 88.6% DPPH reduction), and enhanced epithelialization, angiogenesis, and collagen deposition in full-thickness wounds [194].

Table 2 summarizes representative injectable hydrogels employed in wound healing, highlighting their compositions, key benefits, and the specific healing phases they support.

**Table 2:** Summary of injectable hydrogels for wound healing

Hydrogel type	Composition	Key benefits	Healing-phases supported	References
Gelatin-based hydrogel	Natural polymer derived from collagen	Biocompatibility promotes cell adhesion and proliferation	Hemostasis, Inflammation, Proliferation	[195, 196]
Chitosan-based hydrogel	Natural polymer from chitin	Antibacterial, enhances healing rates, moisture retention	Hemostasis, Inflammation, Proliferation	[28, 193, 197]
Alginic-acid hydrogel	Natural polysaccharide	Biodegradable, forms gel in the presence of calcium	Hemostasis, Inflammation	[198–200]
Hyaluronic-acid hydrogel	Natural polysaccharide	Promotes cell migration and reduces inflammation	Inflammation, Proliferation	[201, 202]
PEG-based hydrogel	Synthetic polymer	Tunable properties, controlled drug release	Inflammation, Proliferation	[203, 204]
PVA-based hydrogel	Synthetic polymer	Mechanical strength, customizable degradation rates	Inflammation, Proliferation	[205, 206]
Thermoresponsive hydrogel	Synthetic/natural combinations	Injectable at room temperature, solidifies in situ	All healing phases depend on the formulation	[207, 208]

### Benefits of injectable hydrogels in wound healing

Injectable hydrogels offer clinically meaningful advantages owing to their tunable biochemical functionality, adaptive mechanics, and ability to form conformal 3D networks in situ [209].

Chitosan-based hydrogels and systems incorporating metallic nanoparticles (e.g., AgNPs) exhibit potent bactericidal and antibiofilm effects while maintaining cytocompatibility. Preclinical in vivo studies report accelerated wound closure, reduced microbial load, and attenuation of infection-associated inflammation following their application to contaminated wound models [210]. Integration of antioxidant moieties—including polyphenols, such as tannic acid—enables efficient ROS scavenging, which is essential for interrupting chronic inflammation cycles. These antioxidant-enriched hydrogels promote angiogenesis, modulate cytokine profiles, and enhance collagen deposition, particularly in diabetic and other impaired-healing environments [211].

Composite hydrogels based on hyaluronic acid, gelatin, or related biopolymers can be engineered for sustained, localized release of growth factors (e.g., VEGF), peptides, and small-molecule therapeutics. Such systems enhance fibroblast proliferation, endothelial cell migration, extracellular matrix synthesis, and neovascularization, ultimately improving wound closure dynamics in vivo [212]. Nanocomposite and dynamically crosslinked designs—incorporating elements, such as lysozyme nanofibrils, gold nanoparticles, or reversible covalent bonds—offer superior injectability, autonomous self-healing, and enhanced mechanical resilience. These architectures can further integrate antioxidant, sensing, or synergistic biological functionalities without compromising biocompatibility [213].

Injectable hydrogels mitigate oxidative stress in chronic wounds through mechanisms that extend beyond antimicrobial activity [214]. By incorporating antioxidant molecules or redox-active components, hydrogel matrices can directly scavenge excessive reactive oxygen species and restore redox balance in inflamed wound environments [215]. In parallel, the physical barrier properties of hydrogels regulate oxygen diffusion and protect regenerating tissue from oxidative damage. Moreover, sustained release of bioactive factors and immunomodulatory cues from injectable hydrogels suppresses pro-inflammatory cell activation and reduces ROS overproduction associated with chronic inflammation and ischemia. These combined effects position injectable hydrogels as dynamic regulators of oxidative stress, supporting tissue regeneration and functional wound repair [42].

### Design considerations for enhanced efficacy

The therapeutic performance of injectable hydrogels depends on strategic optimization of polymer composition, network architecture, stimuli-responsiveness, drug-release kinetics, and adaptability to evolving wound microenvironments. Polymers—natural, synthetic, or hybrid—must be tuned to achieve an appropriate balance between mechanical stability, injectability, degradation rate, and porosity. Nanocomposite designs substantially enhance functional performance. Incorporating nanoparticles into polymer matrices can yield hydrogels with improved mechanical strength, antimicrobial activity, antioxidant capacity, or self-healing behavior. For example, an MC@ZIF-8 nanocomposite hydrogel demonstrated strong tissue adhesion, intrinsic self-healing, potent antibacterial and antioxidant activity, and markedly improved wound closure in vivo [216]. Given the dynamic, multi-stage nature of wound healing, responsive hydrogels capable of reacting to endogenous cues offer major therapeutic advantages. Smart hydrogels leveraging pH, ROS, enzymes, temperature, light, or glucose as triggers can modulate gelation, degradation, or multi-agent release to align with wound status [217, 218]. A ROS-responsive thiolated HA hydrogel crosslinked via disulfide bonds and loaded with AgNPs and curcumin–liposomes simultaneously provided antimicrobial and antioxidant protection, reduced inflammation, enhanced angiogenesis, and accelerated diabetic wound repair [219]. Multinetwork hydrogels—such as GelMA/HA/tannic acid/collagen III systems—combine covalent and dynamic interactions to provide mechanical robustness, reversible remodeling, and significant regenerative efficacy [194]. Similarly, “all-in-one” hydrogels offering multi-therapeutic release have demonstrated strong bactericidal activity and rapid wound closure, highlighting the translational potential of intelligent, stage-specific delivery systems [220].

A dual-responsive hydrogel triggered by low pH and elevated ROS enabled synchronized co-release of amikacin and naproxen specifically within infected and inflamed wound sites, achieving on-demand antimicrobial and anti-inflammatory therapy [221]. Nanohybrid hydrogels incorporating nanozymes or catalytic nanomaterials further expand capability by providing simultaneous antimicrobial, antioxidant, immunomodulatory, and angiogenic functions, making them particularly suited for chronic or infected wounds [222]. Despite progress, translational barriers persist, including challenges in reproducing spatiotemporal wound heterogeneity, achieving precise multi-agent release profiles, scaling complex hydrogel architectures, and ensuring long-term biosafety and regulatory compliance. Addressing these challenges will require standardized design frameworks, scalable manufacturing technologies, and rigorous preclinical testing in clinically relevant wound models [223].

### Spatiotemporal drug delivery challenges in heterogeneous wound microenvironments

Heterogeneous wound microenvironments pose substantial challenges to achieving precise spatiotemporal control over drug delivery, owing to pronounced regional and temporal variations in pH, enzymatic activity, oxygen tension, inflammatory mediators, and cellular composition. Such microenvironmental gradients are especially evident in chronic and infected wounds, where ischemic cores, hypoxic zones, and highly inflamed peripheral regions coexist within a single lesion [224, 225].

Most current injectable hydrogel platforms rely predominantly on single-stimulus responsiveness (e.g., pH- or enzyme-triggered release), which inherently limits their capacity to discriminate between distinct wound niches and to dynamically adapt to evolving pathological conditions. Furthermore, diffusion-dominated drug transport**,** excessive wound exudation, irregular extracellular matrix remodeling, and mechanical deformation of the wound bed collectively contribute to uncontrolled drug redistribution, thereby undermining spatial targeting and therapeutic efficacy [226].

Critically, the lack of integrated feedback mechanisms capable of sensing and responding to real-time changes in the wound microenvironment, together with the scarcity of clinically predictive in vivo models that accurately recapitulate wound heterogeneity, continues to impede the rational design and translational validation of spatially controlled drug delivery systems. These limitations underscore an urgent need for next-generation injectable hydrogels featuring multi-responsive behavior, spatial programmability, and adaptive release profiles that can autonomously tailor therapeutic delivery in response to dynamic wound-specific cues, thereby enabling more effective and personalized wound management strategies [227].

### Clinical translation and health-economic considerations cost-effectiveness considerations

Despite growing clinical interest, the translation of injectable hydrogel technologies into routine wound care practice remains constrained by economic, regulatory, and reimbursement challenges. Injectable hydrogels typically incur higher material, formulation, and manufacturing costs than conventional dressings due to complex polymer synthesis, sterilization requirements, and quality control for injectable formulations. As a result, their clinical adoption depends not on demonstrated therapeutic benefit alone, but on whether these added costs can be justified within existing healthcare payment frameworks. From a health-economic perspective, injectable hydrogels must demonstrate cost–benefit superiority over standard care when evaluated across the full treatment pathway rather than at the point of purchase. Preliminary clinical and modeling studies suggest that reductions in wound-related complications, secondary infections, and treatment escalation may offset higher upfront costs by lowering downstream expenditures, including hospitalization, procedural interventions, and intensive nursing care [227]. However, these conclusions are largely derived from small-scale studies or indirect economic analyses, limiting their generalizability.

A major barrier to definitive economic validation is the scarcity of prospective, standardized health-economic evaluations. Few studies incorporate quality-adjusted life year (QALY) metrics, long-term recurrence rates, or real-world cost data that account for heterogeneity in wound type, patient comorbidities, and healthcare systems. Such data are essential for defining cost-effectiveness thresholds that are acceptable to payers and policy makers, particularly in resource-constrained settings [228–230]. In parallel, reimbursement pathways for injectable hydrogel-based wound therapies remain underdeveloped. Many products do not clearly align with existing procedural or device reimbursement codes, creating uncertainty for clinicians and healthcare providers. Early engagement with regulatory agencies and payers, along with the incorporation of health-economic endpoints into late-stage clinical trials, will be critical to support coverage decisions and large-scale implementation.

## Conclusions and future perspectives

Injectable hydrogels have emerged as one of the most promising classes of biomaterials for advanced wound management, owing to their adaptable physicochemical properties, excellent biocompatibility, and ability to conform to complex tissue geometries. Recent developments in polymer chemistry, crosslinking strategies, and nanocomposite design have significantly expanded their therapeutic potential, enabling multifunctional systems capable of providing structural support, regulating inflammation, combating infection, and promoting tissue regeneration. Despite these advances, translation from laboratory research to clinical practice remains contingent on rigorous standardization, comprehensive characterization, and alignment with regulatory requirements. Ensuring batch-to-batch reproducibility, establishing robust safety profiles, and optimizing large-scale manufacturing processes are essential for bridging the gap between experimental innovation and real-world clinical application. Enhanced analytical techniques, including advanced mechanical, thermal, spectroscopic, and microstructural assessments, will continue to play a pivotal role in defining the quality attributes required for safe and reliable clinical use. Looking ahead, next-generation injectable hydrogels are expected to evolve into highly sophisticated therapeutic platforms. Smart and stimuli-responsive formulations capable of dynamically adjusting to wound microenvironmental cues will allow more precise control over drug release, degradation behavior, and biological activity. Integrating functionalities such as immunomodulation, angiogenic stimulation, and hemostatic enhancement will further elevate their therapeutic impact. Moreover, the convergence of biomaterials science with digital technologies—such as biosensors, real-time monitoring systems, and data-driven personalization—offers an exciting direction for future development, potentially enabling responsive, patient-specific therapies.

Ultimately, the advancement of injectable hydrogels for wound healing will depend on strong interdisciplinary collaboration among materials scientists, clinicians, bioengineers, and regulatory experts. By addressing current translational challenges and embracing emerging technological opportunities, injectable hydrogels are poised to become key components of next-generation regenerative medicine, offering more effective, personalized, and clinically meaningful solutions for complex wound healing.

## Data Availability

Data sharing is not applicable to this article as no new data were created or analyzed in this study.
